# A Granger Causality Measure for Point Process Models of Ensemble Neural Spiking Activity

**DOI:** 10.1371/journal.pcbi.1001110

**Published:** 2011-03-24

**Authors:** Sanggyun Kim, David Putrino, Soumya Ghosh, Emery N. Brown

**Affiliations:** 1 Department of Brain and Cognitive Sciences, Massachusetts Institute of Technology, Cambridge, Massachusetts, United States of America; 2 Department of Anesthesia and Critical Care, Massachusetts General Hospital, Boston, Massachusetts, United States of America; 3 Centre for Neuromuscular and Neurological Disorders, University of Western Australia, QEII Medical Centre, Nedlands, Australia; 4 Division of Health Sciences and Technology, Harvard Medical School/Massachusetts Institute of Technology, Cambridge, Massachusetts, United States of America; University College London, United Kingdom

## Abstract

The ability to identify directional interactions that occur among multiple neurons in the brain is crucial to an understanding of how groups of neurons cooperate in order to generate specific brain functions. However, an optimal method of assessing these interactions has not been established. Granger causality has proven to be an effective method for the analysis of the directional interactions between multiple sets of continuous-valued data, but cannot be applied to neural spike train recordings due to their discrete nature. This paper proposes a point process framework that enables Granger causality to be applied to point process data such as neural spike trains. The proposed framework uses the point process likelihood function to relate a neuron's spiking probability to possible covariates, such as its own spiking history and the concurrent activity of simultaneously recorded neurons. Granger causality is assessed based on the relative reduction of the point process likelihood of one neuron obtained excluding one of its covariates compared to the likelihood obtained using all of its covariates. The method was tested on simulated data, and then applied to neural activity recorded from the primary motor cortex (MI) of a *Felis catus* subject. The interactions present in the simulated data were predicted with a high degree of accuracy, and when applied to the real neural data, the proposed method identified causal relationships between many of the recorded neurons. This paper proposes a novel method that successfully applies Granger causality to point process data, and has the potential to provide unique physiological insights when applied to neural spike trains.

## Author Summary

Recent advances in multiple-electrode recording have made it possible to record the activities of multiple neurons simultaneously. This provides an opportunity to study how groups of neurons form functional ensembles as different brain areas perform their various functions. However, most of the methods that attempt to identify associations between neurons provide little insight into the directional nature of the interactions that they detect. Recently, Granger causality has proven to be an efficient method to infer causal relationships between sets of continuous-valued data, but cannot be directly applied to point process data such as neural spike trains. Here, we propose a novel and successful attempt to expand the application of Granger causality to point process data. The proposed method performed well with simulated data, and was then applied to real experimental data recorded from sets of simultaneously recorded neurons from the primary motor cortex. The results of the real data analysis suggest that the proposed method has the potential to provide unique neurophysiological insights about network properties in the cortex that have not been possible with other contemporary methods of functional interaction detection.

## Introduction

Neurons in the brain are known to exert measurable, directional influences on the firing activities of surrounding neurons, and a detailed analysis of these interactions improves our understanding of how the brain performs specific functions [1]. Attempts to identify associations between neurons, such as the cross-correlogram [2], joint peri-stimulus time histogram [3], smoothed ratio of spiking activity [4], and gravitational clustering [5], have been useful in the past. However, these methods provide little insight into the directional nature of the interactions that they detect, are less reliable in their detection of inhibitory interactions, and usually do not consider the point process nature of neural spike train data. Occasionally they may also give a misleading picture of the relationships between neurons if the detected associations are caused by common inputs or mediated by other neurons [6].

Granger causality has proven to be an effective method for the investigation of directional relationships between continuous-valued signals in many applications [7]–[11]. The basic idea of causality between signals was introduced by Wiener [12] but was too general to be implemented. Granger formalized this idea in order to enable practical implementation based on the multivariate autoregressive (MVAR) models [7]: if past values of 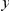 contain information that helps predict 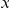 above and beyond the information contained in past values of 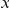 alone, then 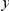 is said to Granger-cause (or g-cause) 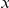. Its mathematical formulation is based on the MVAR modeling of processes. However, it is difficult to apply this method directly to spike train data, since they can not be described by the MVAR model, and standard distance measures such as the mean squared error (MSE) are not designed for spike train data. Recently, several methods have been developed to apply Granger causality to spike train data [13]–[19]. Attempts at transforming neural spike trains into continuous-valued data by convolving spike trains with either a smooth kernel [13] or a lowpass filter [14], [15] have been proposed, but they introduced unwanted distortion of the point process characteristics of spike train data. Granger causality analysis based on an MVAR-nonlinear-Poisson model has been proposed [16]; however, this approach lacks an explanation of the physical meaning of the model that is being applied. A method called transfer entropy using mutual information has also been proposed [17], [18], and it is sensitive to nonlinear signal properties, but unfortunately its application is restricted to bivariate cases. A nonparametric method based on spectral matrix factorization has been proposed [19]; however, it required the second-order stationarity of spike train data.

To address these issues, this paper proposes a point process framework for assessing Granger causality between multiple neurons. The spiking activity of each neuron is simultaneously affected by multiple covariates such as its own spiking history and the concurrent ensemble activity of other neurons. The effect of these factors on a neuron's spiking activity is characterized by a statistical framework based on the point process likelihood function, which relates the neuron's spiking probability to the covariates [20], [21]. Using the point process likelihood function, Granger causality between neurons is assessed based on the likelihood ratio statistic. That is, Granger causality from neuron 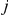 to neuron 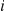 is measured based on the relative reduction of the point process likelihood of neuron 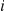 obtained by excluding the covariates corresponding to the effect of neuron 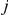 compared to the likelihood obtained using all the covariates. If the likelihood ratio is less than one, we say that there is a causal influence from neuron 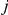 to 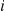, and if the ratio is one, we say that there is no causal influence. In continuous-valued cases, the Granger causality measure based on the MVAR prediction error was shown to be the likelihood ratio test statistic if the prediction error is assumed to be Gaussian [22]. In addition, the point process likelihood ratio statistic enables us to perform statistical hypothesis testing to investigate the significant causal interactions between neurons, since it asymptotically follows a chi-squared distribution when the conditional intensity function (CIF) of the point process is modeled by the generalized linear model (GLM) [23]. When performing a set of statistical inferences simultaneously to detect statistically significant causal interactions among all possible interactions, multiple hypothesis testing problems where the null hypothesis is more likely to be incorrectly rejected should be considered. The present study uses the false discovery rate (FDR) correction to control the expected proportion of incorrectly rejected null hypotheses [24].

The proposed framework was used in an attempt to identify the causal relationships between simulated spike train data, and accurately estimated the underlying causal networks presented in the simulations. It was also applied to real neural data recorded from the cat primary motor cortex (MI) in order to assess the causal relationships that occur between multiple simultaneously recorded neurons during performance of a movement task.

## Methods

### Ethics Statement

The experiments that were performed for the collection of real neural spiking data were approved by the Animal Ethics Committee of the University of Western Australia, and the National Health and Medical Research Council of Australia (NH&MRC) guidelines for the use of animals in experiments were followed throughout.

### Summary of the Proposed Method

Statistical analysis of the potential causal relationships between neurons was performed based on a point process likelihood function. The likelihood function related a neuron's spiking probability to possible covariates, such as its own spiking history and the concurrent activity of all simultaneously recorded neurons. The causal relationships between associated neurons were assessed based on the point process likelihood ratio, which represents the extent to which the likelihood of one neuron is reduced by the exclusion of one of its covariates, compared with the likelihood if all of the available covariates are used. The Granger causality measure based on the point process likelihood ratio also enabled us to detect significant causal relationship through a hypothesis testing based on the likelihood ratio statistic.

### A Granger Causality Measure for Point Process Models

A point process is a time series of discrete events that occur in continuous time [25]. The discrete, all-or-nothing nature of a sequence of action potentials together with their stochastic structure suggests that neural spike trains may be regarded as point processes [20], [26]–[28]. Given an observation interval 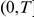, let 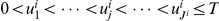 be a set of 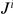 spike times point process observations for 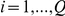 recorded neurons. Let 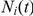 denote the sample path that counts the number of spikes of neuron 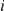 in the time interval 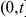 for 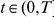. A point process model of a spike train for neuron 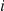 can be completely characterized by its CIF, 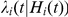, defined as
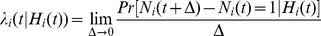
(1)where 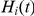 denotes the spiking history of all the neurons in the ensemble up to time 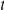 for neuron 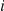 [25]. In this work, 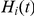 is defined in the interval 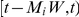, which is divided into 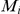 non-overlapping rectangular windows of duration 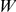; We denote the spike count of neuron 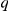 in a time window of length 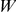 covering the time interval 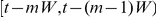 as 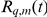 for 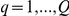 and 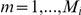. The CIF, 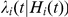, of (1) represents the firing rate of neuron 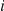 at time 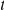, so it quantifies the probability that neuron 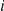 fires a spike at time 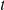 given its covariates 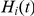. Each neuron has a different 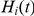, since each has a history dependency of different length, 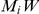. The probability that neuron 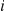 fires a single spike in a small interval 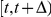 can be approximated as 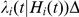.

To model the effect of its own and ensemble's spiking histories on the current spiking activity of a neuron, a GLM framework is often used to model the CIF. In the GLM framework, the logarithm of the CIF is modeled as a linear combination of the functions of the covariates that describe the neural activity dependencies [20], [21]. Thus, the logarithm of the CIF is expressed as
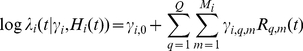
(2)where 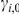 relates to a background level of activity of neuron 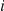, and 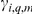 represents the effect of ensemble spiking history 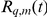 on the firing probability of neuron 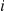. The parameter vector 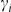 is given as
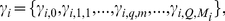
(3)which represents the dependency of neuron 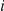 on the spiking history of all neurons in the ensemble. Especially, the parameters 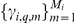 represent the dependency of neuron 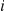 on the spiking history of neuron 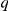 for 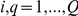. The model for the CIF of (2) is not a fixed form, but can change depending on its covariates and its relationship to them.

A point process likelihood function was used to fit the parametric CIF and analyze Granger causality between neurons since it is a primary tool used in constructing statistical models and has several optimality properties [29]. Here, we used a discrete time representation of the point process likelihood function in order to simplify ensuing calculations. To obtain this representation, we partitioned the observation interval 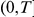 into 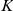 subintervals 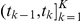 each of length 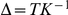 where 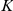 is a large integer. Usually, 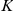 is chosen to make 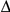 as 1 ms. We denote the continuous time variables defined above as the discrete time versions such as 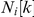 for 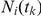, 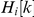 for 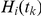, 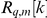 for 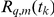 and so forth. Since we chose a large value for 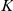, there is at most one spike per subinterval, that is, 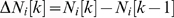 takes on the value 0 if there is no spike in 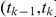 or 1 if there is a spike. The parametric form of the CIF of (2) for neuron 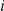 is represented as 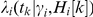.

Given the ensemble spiking activity in 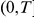, the likelihood function of the spike train of neuron 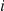 is given as in [20] using its CIF by
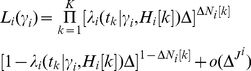
(4)where the term 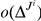 relates the probability that neuron 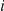 includes two or more spikes in any subinterval 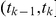. Based on the likelihood function of (4), a point process framework for assessing the causal relationships between neurons is proposed. A potential causal relationship from neuron 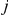 to neuron 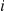 is assessed by calculating the relative reduction in the likelihood of producing a particular set of spike trains of neuron 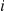 if the spiking history of neuron 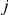 is excluded, compared with the likelihood if all of the available covariates are used. The log-likelihood ratio, 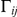, is given by
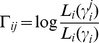
(5)where the likelihood 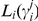 is obtained using a new CIF, 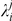, which excludes the effect of neuron 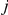 from 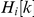, given as
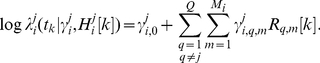
(6)The parameter vector 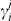 is obtained by re-optimizing the parametric likelihood model after excluding 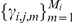 from 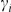 in order to remove the effect of neuron 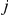 on neuron 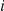, and 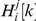 is obtained by leaving out 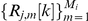 from 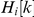. Since the likelihood 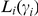 is always greater than or equal to the likelihood 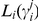, the log-likelihood ratio 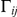 is always less than or equal to 0. If the spiking activity of neuron 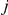 has a causal influence on that of neuron 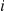 in the Granger sense, the likelihood 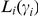 that is calculated using all the covariates of neuron 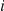 is greater than the likelihood 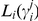 that is calculated using the same covariates, save for the history of neuron 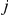, which is excluded. Excitatory and inhibitory influences of neuron 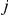 on neuron 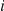 can be distinguished by the sign of 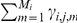 that represents an averaged influence of the spiking history of neuron 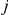 on neuron 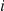. The equality holds when neuron 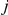 has no influence on neuron 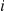. Thus, the Granger causality measure from neuron 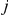 to neuron 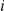 is proposed as
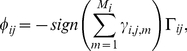
(7)which provides an indication of the extent to which the spiking history of neuron 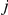 affects the spike train data of neuron 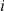. A positive result is indicative of neuron 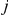 having an excitatory effect upon neuron 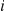, a negative result indicates an inhibitory effect, and zero indicates that no interactions are detected. Finally, a 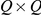 Granger causality matrix can be produced, 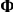, whose 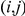
th element is 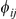, and represents the extent to which neuron 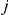 has either an excitatory or inhibitory influence on neuron 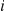 for 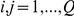.

### Significance Test

The Granger causality matrix 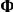 represents the relative strength of estimated causal interactions between neurons, but does not provide any insight into which of these interactions are statistically significant. To address this issue, a hypothesis testing based on the likelihood ratio test statistic is performed to evaluate the statistical significance of the estimated causal interactions of 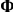. For this, the goodness-of-fit (GOF) statistics are applied as follows [23], [29]. Let us denote the deviance obtained using the model parameter 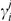 as 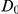 and the deviance obtained using the model parameter 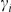 as 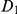; The deviance is obtained by comparing the estimated model with a more general model that has a parameter for every observation so that the data fits exactly, which is called a full model [25], [30]. Its expression is −2 times the log-likelihood ratio of the estimated model to the full model, which is mathematically expressed by
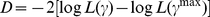
(8)where 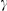 and 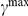 are the parameters for the estimated and the full models, respectively. In the GLM framework the deviance is used to compare two models, which are nested like the above case, since a model of 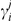 is a special case of the more general model of 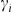. Consider the null hypothesis
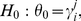
(9)which corresponds to the model of (6). An alternative hypothesis is
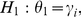
(10)which corresponds to the model of (2).

We can test 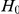 against 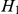 using the difference of the deviance statistic as the test statistic, which is given by
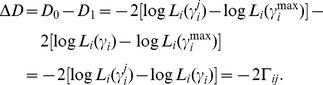
(11)Thus, the deviance difference between two models is equivalent to −2 times log-likelihood ratio given by (5). If both models describe the data well, then the deviance difference may be asymptotically described as 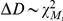 where 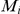 is equal to the difference in dimensionality of the two models [23], [29]. If the value of 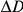 is consistent with the 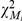 distribution, the hypothesis 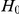 is accepted since it is simpler. This result indicates that the past values of neuron 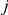 contain no significant information that would assist in predicting the activity of neuron 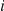. Thus, neuron 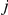 has no causal influence on neuron 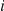. On the contrary, if the value of 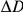 is in the critical region, i.e., greater than the upper tail 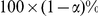 of the 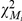 distribution where 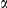 determines false positive rates, then 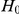 may be rejected in favor of 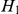 since the model of (2) describes the data with significantly more accuracy. This indicates that past spike times of neuron 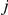 contain information that improves the ability to predict the activity of neuron 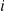. Thus the activity of neuron 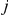 g-causes the activity of neuron 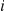.

In any attempt to identify the causal relationships between multiple neurons simultaneously, the total number of the possible causal interactions to be investigated is usually large. Thus, the use of common statistical thresholds cited above to assess the causal interactions would lead to an unacceptably large number of false causal interactions (false positives) where the null hypothesis is incorrectly rejected [31]. The multiple comparison problem could potentially be addressed by the use of stricter statistical thresholds, which would result in a reduction in the proportion of the falsely rejected null hypotheses. However, stricter thresholds would also reduce the probability that true causal interactions between neurons were identified. The present study uses a multiple-hypothesis testing error measure called the FDR to address the multiple comparisons problem. The FDR controls the expected proportion of false positive findings among all the rejected null hypotheses [24]. In situations where the number of hypothesis tests is large, other approaches that attempt to control the familywise error rate (FWER), which is the probability of making one or more false discoveries among all the hypotheses, can be too strict and decrease the power. Thus, the FDR is a less conservative, but more powerful, quantity to control for multiple comparisons than the FWER at a cost of increasing the likelihood of obtaining false positive findings [32].

Combining the multiple hypothesis testing results with the sign of 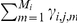, we detect the inhibitory, excitatory, and non-causal interactions, which are denoted as the blue, red, and green colors, respectively. Thus, a 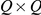 causal connectivity matrix 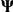 whose 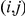
th element corresponds to one of three interactions is constructed. In this paper, the connectivity matrix 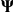 was obtained by controlling the FDR as 0.05.

## Results

### Simulation

**Figure 1:**
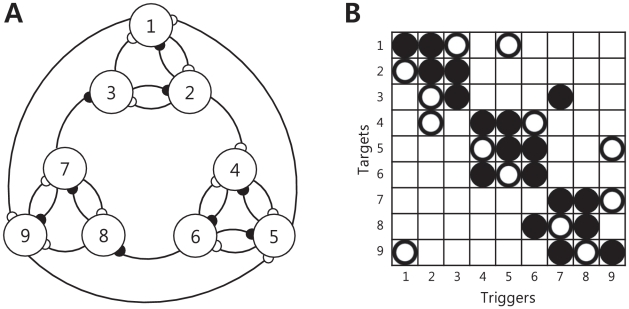
Nine-neuron network to generate synthetic neural spike trains. (A) Each neuron had a spontaneous firing rate 18 Hz and was self-inhibitory. Neurons interact through inhibitory (black) and excitatory (white) connections. The firing probability of each neuron is modulated by a self-inhibitory interaction in addition to the inhibitory and excitatory interactions. (B) The true causal connectivity map is obtained from (A). Black and white circles indicate the respective inhibitory and excitatory influence from trigger neuron to target.

**Figure 2:**
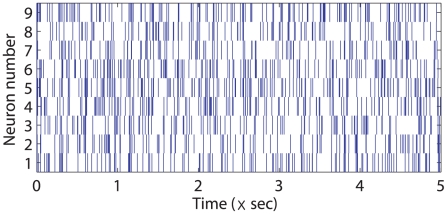
Spike train examples generated based on the nine-neuron network of **Figure 1**. It can be seen that neurons generally fire less (or more) spikes after other neurons with inhibitory (or excitatory) influence on them fire spikes. However, it is hard to estimate the underlying causal network between neurons directly from this plot.

In order to evaluate the proposed framework's ability to identify Granger causality for ensemble spiking activity, we analyzed synthetically generated spike train data. Simulated spike train data were synthetically generated based on the nine-neuron network of Figure 1. The firing probability of each neuron was dependent on its own spiking history and the concurrent ensemble activity through the inhibitory and the excitatory interactions of Figure 1. The inhibitory and the excitatory interactions were represented as black and white circles, respectively. Each neuron was influenced by other neurons through two inhibitory interactions including its own self-inhibition and through one or two excitatory interactions. The overall network of Figure 1A consisted of three sub-networks each with three neurons. The interactions between neurons within sub-networks were set to have relatively small duration, and the parameter vectors for the inhibitory and the excitatory interactions among neurons were set to 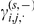 = [−0.8 −0.6 −0.3] and 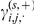 = [1 2 2], respectively. For interactions between different sub-networks, the parameter vectors for the inhibitory and excitatory interactions were set to have relatively long duration such as 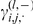 = [0 0 0 −0.8 −0.9 −0.5] and 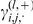 = [0 0 0 1 2 1], respectively. The parameter vector for the self-inhibition was set to 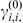 = [−0.6 −0.5 −0.4]. All neurons had the same spontaneous firing rate (18 Hz). Spike trains for neuron 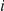 were generated using a commonly used procedure as follows [33]: A random number 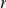, uniformly distributed between 0 and 1, is generated at every interval; if 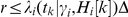, a spike is presumed to have occurred in 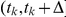; otherwise, no spike is generated. The time resolution 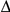 was set to 1 ms. An absolute refractory period of 1 ms was enforced to prevent neurons from firing a spike in adjacent time steps. Based on the experimental settings cited above, we generated 100,000 samples for each neuron, and the total number of spikes for each neuron ranged from 2176 through 2911. Examples of generated neural spike trains during the first 5 sec (5,000 samples) are illustrated in Figure 2. It can be seen that neurons generally fire less (or more) spikes after other neurons with inhibitory (or excitatory) influence on them fire spikes. However, it is hard to estimate the underlying causal network between neurons from this plot.

In order to select a model for each neuron we fit several models with different history durations 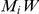 to each spike train data and then identified the best approximating model from among a set of candidates using Akaike's information criterion (AIC) [34], [35]. Using this criterion, an optimum model order for each neuron was selected. The spike counting window length 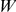 was set to 2 ms. For neurons 1, 3, 4, 5, 8, and 9, which were influenced by other neurons through relatively long interactions, the selected GLM spike order 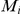 was 3, which indicates a 6 ms history duration, and for neurons 2, 6, and 7 influenced by other neurons within same sub-network only through short interactions, the selected GLM order 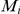 was 2, which corresponds to a 4 ms history duration.

**Figure 3:**
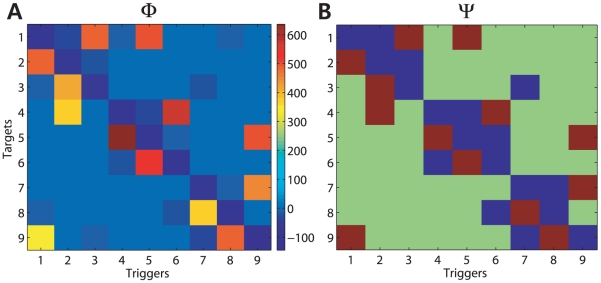
Two causality maps, 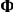 and 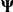, estimated using the simulated data. (A) The Granger causality matrix 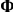 shows how much each neuron interacts one another. (B) The causality connectivity map 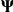 was obtained through the hypothesis testing. As shown, the red and blue colors denote the presence of the inhibitory and the excitatory interactions from trigger neuron to target, respectively. The green color represents that there is no causal interaction between the tested neurons. The estimated pattern matches the actual network of Figure 1.

Based on the estimated model, two kinds of causality maps were obtained using the proposed method. Firstly, the Granger causality map 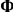, which is illustrated in Figure 3A, represents the relative strength of the causal interaction between neurons. It represents the extent to which a trigger neuron has a causal impact on a target compared to other interconnections, but provides little insight into which causal impact is statistically significant. In order to make up for 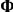, the causal connectivity map 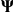 was obtained through the hypothesis testing when we controlled the FDR as 0.05. This is shown in Figure 3B. The red, blue, and green colors denote the presence of excitatory, inhibitory, or no interactions from trigger neuron to target, respectively. The estimated pattern of 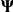 matches the actual network of Figure 1 exactly. This causality map does not show a connection between neurons that do not have direct interactions, even though they have indirect interactions.

**Figure 4:**
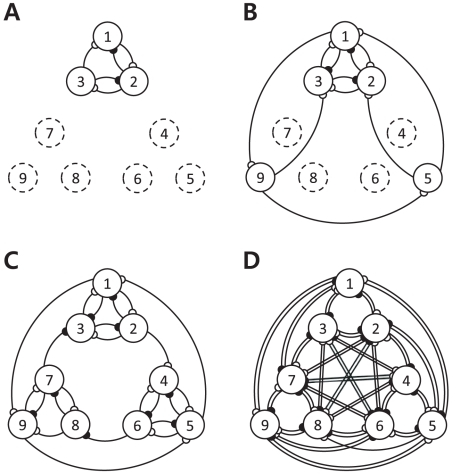
Four different networks used to generate synthetic neural spike trains. (A) A nine-neuron network with 8 causal interactions is illustrated. All experimental conditions remained the same as those that were used for the production of Figure 1. The dashed circle represent a neuron that has no self-interaction effect on itself. (B) A nine-neuron network with 16 interactions is illustrated. (C) A nine-neuron network with 32 interactions is illustrated. (D) A nine-neuron network with 64 interactions is illustrated.

**Figure 5:**
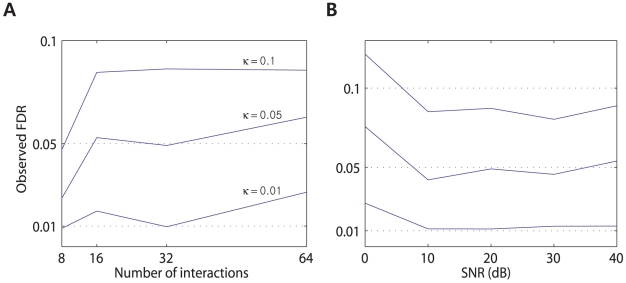
Monte-Carlo simulations performed in order to verify the control of the FDR. (A) The observed FDR is illustrated for 8, 16, 32, and 64 causal interactions at the significance levels of 0.01, 0.05, and 0.1. (B) The observed FDR is illustrated by varying the SNR from about 0 to 40 dB at the significance levels of 0.01, 0.05, and 0.1.

The FDR procedure was used as a solution for the multiple comparisons problem when considering a set of statistical inferences simultaneously. When controlling the FDR at a specific significance level 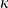, we expect that on average there will be 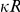 false positives amongst 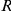 detected significant interactions. In order to verify that the FDR is actually being controlled at the significance levels that we are claiming in the present study, the Monte-Carlo (MC) simulations were conducted by varying both the number of causal interactions between the virtual neurons, and the signal-to-noise ratio (SNR) of the simulated spikes. These MC simulations show how effectively the FDR is being controlled under different experimental conditions. Firstly, we conducted a series of the MC simulations by changing the number of causal interactions from 8 to 64. Data was synthetically generated to resemble four different kinds of networks (seen in Figure 4), each having a different incidence of interaction between neurons. Fifty data sets were generated for each network condition, while all other experimental parameters remained the same. The dashed circle in Figure 4A and B represents a neuron whose firing activity does not depend on the spiking history, and thus follows a homogeneous Poisson process, i.e., 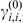 = 0. Networks of Figure 4A to D consist of 8, 16, 32, and 64 interactions (including self-interactions), respectively. The observed FDR is calculated by averaging the ratio of the number of false positives to the number of detected significant interactions over 50 simulations, and it is illustrated in Figure 5A for significance levels of 0.01, 0.05 and 0.1. The FDR was generally controlled at the significance level that we were attempting to control except for the 8-interaction case with less false positives than the number expected at that significance level. We then performed another MC simulation by changing the SNR. Noisy neural spike trains were generated using the CIF of (2) in the following: We added a Gaussian noise to the logarithmic CIF, i.e., the right-hand side of (2), and then generated spike trains using the perturbed CIF. The noise changed the background level of firing rate over time. The SNR is defined as the ratio between the unperturbed logarithmic CIF and the perturbation itself. Fifty data sets of noisy spike trains were synthetically generated based on the nine-neuron network of Figure 1 with different levels of noise, which led to about 0, 10, 20, 30, and 40 dB SNRs, respectively. All other experimental conditions are same to the previous case. Figure 5B illustrates the simulation results obtained for significance levels of 0.01, 0.05, and 0.1. When the SNR is approximately 0 dB, more false positive events were detected than what was expected at the specified significance level, but in most cases the observed FDR was no different from the theoretical FDR. In summary, unless the perturbation level is similar to or higher than the level of the logarithmic CIF of (2) that is modulated by the intrinsic dynamics of the neurons, the FDR is effectively controlled at the significance level that we are attempting to control.

### Real Data Analysis

**Figure 6:**
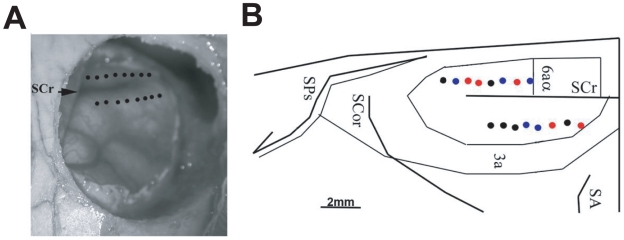
Location of recording electrodes in motor cortex. (A) A peri-operative photograph of a craniotomy, and the locations on the cortical surface where the microwires were implanted (black dots). Rostral is up. (B) A pictorial reconstruction of the unfolded cortical surface, cytoarchitectonic boundaries and electrode locations was created from serial coronal sections. Medial is up. The recording sites where the neurons were simultaneously recorded are labeled in color code. Blue, red and black circles represent sites where one, two or no neurons were able to be recorded, respectively. Abbreviations: 3a, 4 and 6a are cytoarchitectonic areas. Sulci: SA: Ansate sulcus, SCor: Coronal sulcus, SCr: Cruciate sulcus, SPs: Presylvian sulcus.

To illustrate the application of the proposed method to real spike train data, 15 neurons were simultaneously recorded from the cat MI shown in Figure 6 and analyzed. The experimental methodology that was implemented to collect the neural activity used for the following analysis was described in detail in Ghosh et al. [36]. Briefly, an adult cat was trained to perform a skilled reaching movement, using its preferred forelimb to retrieve food pellets placed between 2 upright Perspex barriers spaced 4 cm apart. After behavioral training was complete, PTFE coated Platinum-Iridium microwires were implanted into the cortex to a depth of about 1.5 mm into forelimb and hindlimb representations of MI (identified using intracortical microsimulation). Neural recordings were made as the animal performed the reaching task, and only neurons that significantly modulated their firing rate during task performance were isolated for analysis in this study. Interspike interval, spike duration and spiking rate analyses were performed on neurons isolated from adjacent recording sites. This was done in order to rule out the possibility of the same neuron being counted more than once due to cross-talk between neighboring electrodes. Autocorrelogram, interspike interval and ‘burst surprise’ (using a surprise value of 3) analysis were performed on all neurons in order to identify any potentially bursting neurons in the data set (there were none) [37]–[39]. The data set includes 150,000 samples (3,000 samples/trial
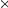
50 trials) for each channel, and the total number of spikes for each neuron across all trials ranged from 613 to 5716. The sampling rate was 1 KHz.

**Figure 7:**
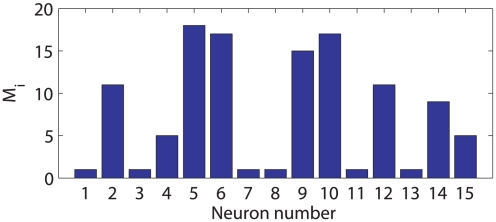
Selected GLM spike history order 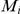 by AIC. Each neuron used 1 parameter (3 ms) through to 18 parameters (54 ms) to model its interconnection.

Using the AIC, an optimum model for each neuron is selected to minimize the criterion. The non-overlapping spike counting window 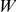 was intuitively set to 3 ms to obtain a relatively small number of parameters while maintaining the temporal resolution. Figure 7 shows the selected GLM spike order 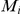 of each neuron for 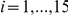, and for each neuron 1 parameter (3 ms) through to 18 parameters (54 ms) were used to model its interconnection.

**Figure 8:**
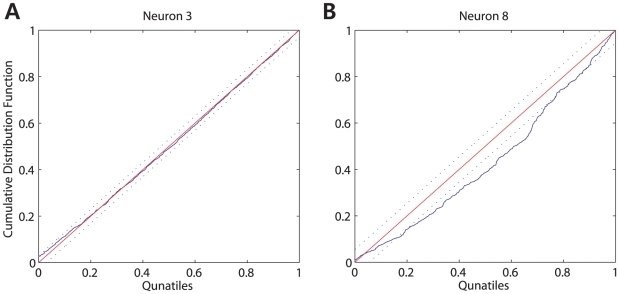
Best and worst KS plots across all 15 neurons. The 45-degree solid line represents exact agreement between the model and spike train data. The two 45-degree dashed lines are the 95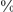 confidence bounds based on the distribution of the KS statistic. (A) Neuron 3 had the best KS plot. (B) Neuron 8 had the worst KS plot.

The GOF of the estimated model is assessed by using the Kolmogorov-Smirnov (KS) plots [40]. Prior to making inferences from an estimated statistical model, it is crucial to measure the agreement between a statistical model and the spike train data. For continuous-valued data, the GOF of the model can be quantitatively measured as standard distance such as MSE. However, this distance measure can not be applied to neural spike train data. To address this problem, we utilized the previously proposed time-rescaling theorem to transform point process measures such as neural spike train data to a continuous measure appropriate for a GOF assessment [40]. Once a CIF is estimated, rescaled times can be computed using the estimated CIF. These rescaled times will be uniformly distributed random variables on the interval 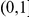 if the estimated CIF is a good approximation to the true conditional intensity of the point process. To evaluate whether the rescaled times follow the uniform distribution, we order these rescaled times from the smallest to the largest, and then plot the quantiles of the cumulative distribution function of the uniform distribution on 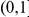 against the ordered rescaled times. This form of graphical representation is termed a KS plot. If the model is consistent with the data, then the points should lie on a 45-degree line. Approximate 95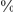 confidence bounds for the degree of agreement between the model and the data may be constructed using the distribution of the KS statistic [41]. Figure 8 shows the best and the worst KS plots obtained using estimated GLMs across all the given spike train data. Most KS plots were almost within the confidence intervals, which indicates that most estimated GLMs fit the data well.

**Figure 9:**
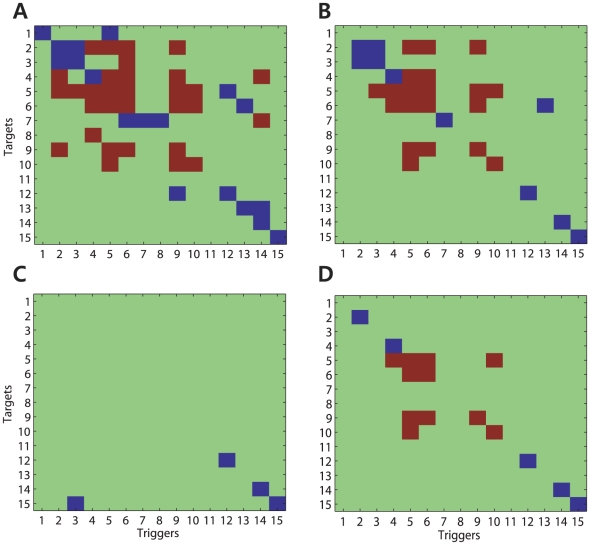
Causal connectivity maps between recorded neurons. (A) The causal connectivity map, 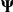, estimated without the application of the multiple comparisons correction is illustrated. (B) The causal connectivity map with the FDR correction is illustrated. (C) The causal connectivity map estimated from another data set recorded during a period of postural maintenance is illustrated. (D) The causal connectivity map was estimated using a smaller number of trials of the reaching task so that the number of spikes is similar to that of data set recorded during postural maintenance.

The causal connectivity between the recorded neural spike train data was assessed using the proposed framework, and the results are illustrated in Figure 9. Figure 9A and B show the causal connectivity maps, 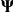, estimated using the proposed framework without and with the FDR correction, respectively. As illustrated in Figure 9A when the multiple comparison problem was not considered, more causal connectivity was estimated; however, there was a high probability that the false rejection of the null hypotheses of the multiple comparison caused the extra causal relationships. In the present study, 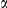 for the hypothesis testing was set to 0.05. After the FDR correction for the multiple comparison problem, the incidence of interactions between the recorded neurons was sparser, and is shown in Figure 9B. In Figure 9B, neurons 2, 3, 4, 5, 6, 9, and 10 exchanged causal interactions with a handful of other neurons including themselves, neurons 7, 12, 14, and 15 showed purely self-inhibitory interactions, and finally neurons 1, 8, 11, and 13 did not receive any influence from other neurons, nor did they show signs of self-interaction. Interestingly, neurons 5, 6, 9, and 10 appeared to display evidence of self-excitatory interactions, which is highly unusual behavior for a neuron. Interspike interval and autocorrelogram analysis were performed on these neurons in order to exclude the possibility that these interactions were occurring due to bursting behavior [37], [38]. Further analysis of these neurons revealed that they also had the four highest history orders among neurons as shown in Figure 7. Figure 9C shows the connectivity map obtained using the neural spike train data recorded during a period of postural maintenance from the same recording sites in MI following completion of a satisfactory number of task trials shown in Figure 6. The data set includes 54,000 samples (3000 sampless/trial
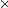
18 trials), and the total number of spikes for each neuron across all trials ranged from 55 to 1030. As shown in the figure, during the state of postural maintenance, most neurons did not show any evidence of significant interactions. It could be argued that the decrease in the number of detected significant interactions that were seen during the state of postural maintenance was actually related to the decreased number of spikes that were observed during this behavioral period. In order to prove that this decrease is actually related to a physiological phenomenon rather than a decreased spike count, causality analysis was performed using the first 11 trials (of a total of 50) of the ‘reaching’ data set, which decreased the averaged number of spikes in that set of data (522 spikes) to a similar level as the ‘postural maintenance’ set (520 spikes). Figure 9D illustrates the obtained causal connectivity map, and more significant interactions were still seen between neurons during reaching movement than during postural maintenance. Note that the obtained causal connectivity maps do not necessarily represent interactions as a result of direct anatomical connection, but suggests that a functional causal connectivity exists between the recorded neurons.

**Figure 10:**
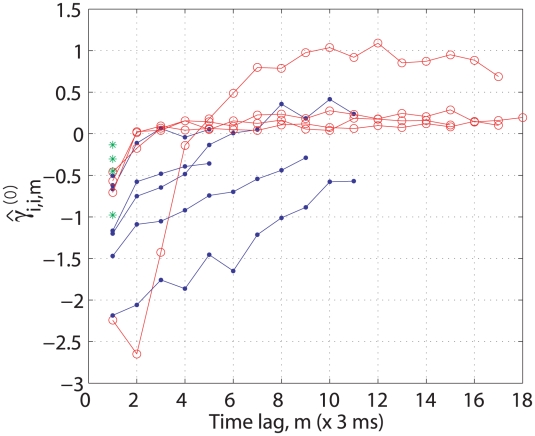
Estimated GLM parameters for self-interactions. The estimated GLM parameters for self-interactions are plotted for all neurons. The red, blue, and green colors denote the excitatory, inhibitory, or no self-interactions, respectively.

The estimated GLM parameters 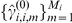 that correspond to the self-interactions of all neurons 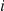 for 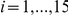 are illustrated in Figure 10. The red, blue, and green colors represent the excitatory, inhibitory, or no self-interactions, respectively. In all cases, the first parameter is always negative due to the absolute refractory period, and the remaining parameters generally have positive values for the self-excitatory interactions and negative values for the self-inhibitory interactions, respectively. In cases where no-interactions was occurring, only one negative parameter (indicated with green asterisk) existed. Neurons showing evidence of excitatory self-interactions have the four highest history orders, and those indicating inhibitory self-interactions have higher orders than those with no self-interactions, which have only one parameter.

## Discussion

We proposed a point process framework for identifying causal relationships between simultaneously recorded multiple neural spike train data. Granger causality has proven to be an effective method to test causality between signals when using the MVAR model, but to date it has been used for continuous-valued data [7]–[11]. The method described in this study represents a novel attempt to apply Granger causality to point process data. The high level of accuracy that our method displayed when predicting the nature of the interactions occurring in the simulated data set was an encouraging indication that the proposed method is sound. Furthermore, the marked disparity in incidence of interactions during movement and non-movement periods in the experimental data is in keeping with the findings of previous studies investigating interactions in MI [36]. Thus, the outcome of both our simulated and experimental data analysis provides compelling evidence that Granger causality can be successfully applied to point process data. This is an important finding, as there are currently very few techniques that assess interactions between multiple neurons as well as providing insight regarding the causal relationships that exist between them. The ability to infer causal relationships between interacting neurons provides us with important information about networks of neurons being studied with this method. A detailed understanding of the interactions occurring in ensemble activity recorded from MI may lead to improved accuracy in algorithms used to control devices such as brain-computer interfaces and neural prosthetics [20], [42], [43].

Other model-based methods for assessing the directional relationships between neurons have been recently developed [21], [42]. These methods infer underlying interactions between neurons based on estimated model parameters, which contain the information on the dependencies between all of the recorded neurons. Thus, functional connectivity between neurons is inferred when the estimated model parameters achieve non-zero magnitude, that is, when their confidence intervals do not cross the zero-magnitude line. However, no quantitative criteria currently exists to guide users of these methods to accept or reject detected interactions when the suspected interaction is of low magnitude, or high magnitude but with wide confidence intervals for the estimated parameters. Thus, in more difficult cases where a model-based method produces uncertain results, the acceptance of a spurious interaction, or the rejection of a legitimate one, may compromise the reliability of experimental data analysis. The proposed point process framework addresses this issue by performing statistical significance tests that investigate the causal interactions based on the likelihood ratio statistic, eliminating this uncertainty. Thus, the proposed method may be of use to researchers who are having trouble quantifying some of the connections that they are detecting when using other model-based methods.

Some of the neurons included in this analysis showed no evidence of either self-interaction, or interactions with other neurons. Although these neurons also had non-zero GLM parameters for self-interaction, as indicated with green asterisks in Figure 10, their effects were tested to be statistically insignificant compared to those caused by other neurons or background firing activity. In these cases, we must consider that the hypothesis testing to evaluate the significant causal interactions depends on the FDR value that is chosen. Decreasing the FDR value means that a statistical threshold for the significance test is more strict, and would lead to a sparser causal connectivity map. Therefore, it should be noted that the inferred causal connectivity maps 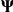 generated by this method are not absolute, and may change depending on the user's selection of the FDR value.

**Figure 11:**
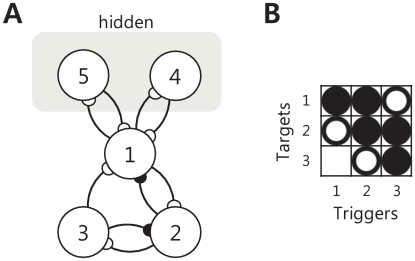
Five-neuron ensemble with a hidden positive feedback network. (A) Five neurons interacting with one other were simulated, but only neurons 1, 2, and 3 were treated as spike trains that were simultaneously recorded together. The activities of neurons 4 and 5 were used to create a hidden positive feedback network with neuron 1, but were treated as neurons outside the receptive field of an electrode: that is, their activities were not used as covariates to create the connectivity maps. (B) The true causal connectivity map between neurons 1, 2 and 3 is obtained from (A).

**Figure 12:**
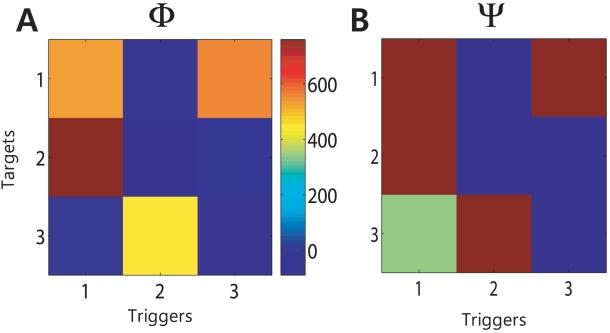
Two causality maps obtained using the simulated data with hidden positive feedback network. (A) The Granger causality matrix 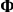 shows the relative strength of causal interactions. (B) The causality connectivity map 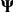 shows the statistically significant causal interactions. The estimated pattern matches the actual network of Figure 11 except that neuron 1 was estimated to have an excitatory self-interaction due to the hidden positive feedback network.

The identification of excitatory self-interactions for some of the analyzed neurons was an unexpected and interesting finding. Analysis of the spiking features of these neurons verified that they were not engaged in any manner of bursting behavior that may explain the self-excitation result. Based on the high history orders that were also seen in those neurons as shown in Figure 10, we infer that the self-excitation result may be caused by ‘hidden’ positive feedback networks, that is, networks involving neurons that were not recorded by our microwires. To support our inference, we have performed another simulation to investigate the effect of hidden feedback networks. We identified the causal interactions among ensemble spiking activity, which was synthetically generated based on the five-neuron network of Figure 11. Compared to the nine-neuron network of Figure 1A, the five-neuron network of Figure 11A had hidden neurons 4 and 5, which composed hidden positive feedback networks together with neuron 1. So the firing activity of neuron 1 was not only dependent on the spiking activity of observed neurons 2 and 3, but also on the spiking activity of hidden neurons 4 and 5; however, only neurons 1, 2, and 3 were observable. The parameter vector for the excitatory interaction of the hidden network was set to 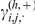 = [0 0 1 2 2 1]. The other experimental settings were all same to the previous case. We generated 100,000 samples for each neuron, and the total number of spikes for the observed neurons 1, 2, and 3 were 4247, 2606, and 2314, respectively. Due to the hidden positive feedback, neuron 1 fired more spikes than other neurons. The model orders were selected using the AIC, and the selected orders for neurons 1, 2, and 3 were 5 (10 ms history duration), 2 (4 ms), and 2 (4 ms), respectively. Neuron 1 had a relatively longer history duration than other neurons due to the hidden feedback networks. Using the proposed method, we obtained both the Granger causality map 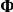 and the causal connectivity map 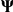, which is illustrated in Figure 12. The estimated causality map 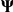 matches well the original network of Figure 11 except that neuron 1 was estimated to have a self-excitatory interaction, which was caused by the hidden positive causal interactions with neurons 4 and 5. This hidden interaction also led to the relatively long history duration of neuron 1 compared to the other neurons. This simulation supported the idea that hidden positive feedback network leads to the relatively long history duration and can change inhibitory self-interaction to excitatory one, which we could also observe in this real data analysis case. Similarly, self-inhibitory interactions, which had a relatively long history duration as shown in Figure 10, were also identified in this study, and may be the result of hidden negative feedback networks. Self-inhibitory interactions (as they are defined using this method) may be difficult to quantify in some cases, as a neuron with a very low firing rate may produce a self-inhibitory result that is similar in appearance to that which would occur due to hidden negative networks. However, the majority of the neurons in the present study that showed the evidence of self-inhibition had quite high firing rates. Thus, the inference of hidden negative feedback networks is a plausible explanation in these cases. The proposed framework creates an unprecedented opportunity to investigate interactions from hidden neural networks that have either excitatory or inhibitory causal influences on recorded neurons. Recently a method called partial Granger causality to identify the underlying causal interactions in the presence of exogenous inputs and latent variables for the continuous-valued case has been proposed [44], [45]. It would be useful to extend this work to neural spike train data in order to deal with the effects of exogenous inputs or hidden neurons beyond the investigation of the hidden feedback network.

The Matlab software and the data sets used to implement the methods presented here are available at the website (http://www.neurostat.mit.edu/gcpp).

## References

[1] Brown EN, Kass RE, Mitra PP (2005). Multiple neural spike train data analysis: state-of-the-art and future challenges.. Nat Neurosci.

[2] Brody CD (1999). Correlations without synchrony.. Neural Comput.

[3] Gerstein GL, Perkel DH (1969). Simultaneously recorded trains of action potentials: analysis and functional interpretation.. Science.

[4] Ventura V, Cai C, Kass RE (2005). Statistical assessment of time-varying dependency between two neurons.. J Neurophysiol.

[5] Gerstein GL, Perkel DH, Dayhoff JE (1985). Cooperative firing activity in simultaneously recorded populations of neurons: detection and measurement.. J Neurosci.

[6] Stevenson IH, Rebesco JM, Hatsopoulos NG, Haga Z, Miller LE (2009). Bayesian inference of functional connectivity and network structure from spikes.. IEEE Trans Neural Syst Rehabil Eng.

[7] Granger CWJ (1969). Investigating causal relations by econometric models and cross-spectral methods.. Econometrica.

[8] Brovelli A, Ding M, Ledberg A, Chen Y, Nakamura R (2004). Beta oscillations in a large-scale sensorimotor cortical network: directional influences revealed by Granger causality.. Proc Natl Acad Sci U S.

[9] Ding M, Chen Y, Bressler SL, Schelter S, Winterhalder N, Timmer J (2006). Grange causality: basic theory and application to neuroscience.. Handbook of time series anal.

[10] Seth AK (2005). Causal connectivity of evolved neural networks during behavior.. Netw Comput Neural Syst.

[11] Chen Y, Bressler SL, Ding M (2006). Frequency decomposition of conditional Granger causality and application to multivariate neural field potential data.. J Neurosci Methods.

[12] Wiener N, Beckenbach EF (1956). The theory of prediction.. Modern mathematics for engineers.

[13] Sameshima K, Baccala LA (1999). Using partial directed coherence to describe neuronal ensemble interactions.. J Neurosci Methods.

[14] Kaminski M, Ding M, Truccolo W, Bressler SL (2001). Evaluating causal relations in neural systems: Granger causality, directed transfer function and statistical assessment of sifnificance.. Biol Cybern.

[15] Zhu L, Lai Y, Hoppensteadt FC, He J (2003). Probing changes in neural interaction during adaptation.. Neural Comput.

[16] Krumin M, Shoham S (2010). Multivariate autoregressive modeling and Granger causality analysis of multiple spike trains.. Comput Intell Neurosci.

[17] Schreiber T (2000). Measuring information transfer.. Phys Rev Lett.

[18] Pereda E, Quiroga RQ, Bhattacharya J (2005). Nonlinear multivariate analysis of neurophysiological signals.. Prog Neurobiol.

[19] Nedungadi AG, Rangarajan G, Jain N, Ding M (2009). Analyzing multiple spike trains with nonparametric Granger causality.. J Comput Neurosci.

[20] Truccolo W, Eden UT, Fellows MR, Donoghue JP, Brown EN (2005). A point process framework for relating neural spiking activity for spiking history, neural ensemble and extrinsic covariate effects.. J Neurophysiol.

[21] Okatan M, Wilson MA, Brown EN (2005). Analyzing functioanal connectivity using a network likelihood model of ensemble neural spiking activity.. Neural Comput.

[22] Geweke J (1982). Measurement of linear dependence and feedback between multiple time series.. J Am Stat Assoc.

[23] Dobson AJ (2002). An Introduction to generalized linear models.

[24] Benjamini Y, Hochberg Y (1995). Controlling the false discovery rate: a practical and powerful approach to multiple testing.. J R Stat Soc Series B Stat Methodol.

[25] Daley DJ, Vere-Jones D (2003). An Introduction to the Theory of Point Process.

[26] Brown EN, Chow C (2005). Theory of point processes for neural systems.. Methods and models in neurophysics.

[27] Brown EN, Barbieri R, Eden UT, Frank L, Feng L (2003). Likelihood methods for neural spike train data analysis.. Computational neuroscience: A comprehensive approach.

[28] Kass RE, Ventura V, Brown EN (2006). Statistical issues in the analysis of neuronal data.. J Neurophysiol.

[29] Pawitan Y (2001). In all likelihood: statistical modeling and inference using likelihood.

[30] McCullagh P, Nelder JA (1989). Generalized linear models.

[31] Miller RG (1981). Simultaneous statistical inference.

[32] Storey JD (2002). A direct approach to false discovery rates.. J R Stat Soc Series B Stat Methodol.

[33] Koch C (1999). Biophysics of computation.

[34] Akaike H (1974). A new look at the statistical model identification.. IEEE Trans Automat Contr.

[35] Burnham KP, Anderson DR (1998). Model selection and inference. A practical information-theoretic approach.

[36] Ghosh S, Putrino D, Burro B, Ring A (2009). Patterns of spatio-temporal correlations in the neural activity of the cat motor cortex during trained forelimb movements.. Somatosens Mot Res.

[37] Bartho P, Hirase H, Monconduit L, Zugaro M, Harris KD (2004). Characterization of neocortical principal cells and interneurons by network interactions and extracellular features.. J Neurophysiol.

[38] Nowak LG, Azouz R, Sanchez-Vives MV, Gray CM, McCormick DA (2003). Electrophysiological classes of cat primary visual cortical neurons in vivo as revealed by quantitative analyses.. J Neurophysiol.

[39] Legendy CR, Salcman M (1985). Bursts and Recurrences of bursts in the spike trains of spontaneously active striate cortex neurons.. J Neurophysiol.

[40] Brown EN, Barvieri R, Ventura V, Kass RE, Frank LM (2002). The time-rescaling theorem and its application to neural spike train data analysis.. Neural Comput.

[41] Johnson NL, Kotz S (1970). Distributions in statistics: continuous univariate distributions 2.

[42] Pillow JW, Shlens J, Paninski L, Sher A, Litke AM (2008). Spatio-temporal correlations and visual signalling in a complete neuronal population.. Nature.

[43] Hatsopoulos N, Harrison M, Donoghue JP (2001). Representations based on neuronal interactions in motor cortex.. Prog Brain Res.

[44] Guo S, Seth AK, Kendrick KM, Zhou C, Feng J (2008). Partial Granger causality-Eliminating exogenous inputs and latent variables.. J Neurosci Methods.

[45] Guo S, Wu J, Ding M, Feng J (2008). Uncovering interactions in the frequency domain.. PLoS Comp Biol.

